# Perception of group membership from spontaneous and volitional laughter

**DOI:** 10.1098/rstb.2020.0404

**Published:** 2022-01-03

**Authors:** Roza G. Kamiloğlu, Akihiro Tanaka, Sophie K. Scott, Disa A. Sauter

**Affiliations:** ^1^ Department of Psychology, University of Amsterdam, REC G, Nieuwe Achtergracht 129B, 1001 NK, Amsterdam, The Netherlands; ^2^ Department of Psychology, Tokyo Woman's Christian University, Tokyo, Japan; ^3^ Institute of Cognitive Neuroscience, University College London, London, UK

**Keywords:** group identity, laughter, spontaneous, voice, volitional

## Abstract

Laughter is a ubiquitous social signal. Recent work has highlighted distinctions between spontaneous and volitional laughter, which differ in terms of both production mechanisms and perceptual features. Here, we test listeners' ability to infer group identity from volitional and spontaneous laughter, as well as the perceived positivity of these laughs across cultures. Dutch (*n* = 273) and Japanese (*n* = 131) participants listened to decontextualized laughter clips and judged (i) whether the laughing person was from their cultural in-group or an out-group; and (ii) whether they thought the laughter was produced spontaneously or volitionally. They also rated the positivity of each laughter clip. Using frequentist and Bayesian analyses, we show that listeners were able to infer group membership from both spontaneous and volitional laughter, and that performance was equivalent for both types of laughter. Spontaneous laughter was rated as more positive than volitional laughter across the two cultures, and in-group laughs were perceived as more positive than out-group laughs by Dutch but not Japanese listeners. Our results demonstrate that both spontaneous and volitional laughter can be used by listeners to infer laughers’ cultural group identity.

This article is part of the theme issue ‘Voice modulation: from origin and mechanism to social impact (Part II)’.

## 1. Introduction

Laughter is a frequently occurring and socially potent nonverbal vocalization, which is frequently used to signal affiliation, reward or cooperative intent, and often helps to maintain and strengthen social bonds [1,2]. A key distinction is whether laughs are spontaneous or volitional [3,4]. Spontaneous and volitional laughs are thought to be generated by different vocal production mechanisms. We often laugh spontaneously with little volitional control, which is thought to typically reflect an internal emotional state. Yet laughter can also be produced with volitional modulation of vocal output, which is more likely to express polite agreement in conversation [5,6]. Recent research has shown that listeners' ability to differentiate individual speakers is impaired for spontaneous, as compared to volitional, laughter [7,8]. Identity-related information is thus decoded more successfully from volitional laughter cues, that is, laughter that was produced with greater vocal control. Here, we build on this work to examine whether laughter type influences the identification not only of individuals, but also of groups. Specifically, we test the prediction that it is easier to identify group membership from volitional as compared to spontaneous laughter. We further explore how the perceived positivity of laughter differs between the two types of laughter, as well as in relation to perceived group membership.

### (a) Spontaneous versus volitional laughter

Spontaneous and volitional laughter differ in terms of how they are produced. Spontaneous laughter is generated by an evolutionarily conserved vocal production system, and is homologous to play vocalizations in nonhuman primates [9]. Producing spontaneous laughter thus requires little volitional control and minimal supralaryngeal modulation, as the articulators are mostly in their resting positions [10]. Volitional laughter, in contrast, involves more complex coordination of articulators and thus requires greater volitional control. Such flexible modulation of the voice is particularly pronounced in human vocal production as compared to other primates [11]. Neurobiological accounts suggest that spontaneous laughter is under the control of an evolutionarily ancient midline system associated with innate vocalizations, while the production of volitional laughs is controlled by regions of the lateral motor cortex associated with learned vocalizations like speech [12].

Evidence suggests that laughter that is generated via volitional vocal production encodes information about the producer more reliably than does spontaneous laughter. Specifically, the discrimination of speaker identity is better for volitional as compared to spontaneous laughter, a pattern found for both familiar and unfamiliar speakers [7]. Notably, the main factor driving the enhanced speaker identity perception from volitional laughter was not *perceptual* properties like authenticity as judged by listeners (i.e. how fake or genuine a laugh sounds), but whether the laughs were *produced* spontaneously or volitionally [8]. This greater encoding of identity-related information in volitionally produced laughter suggests that humans may produce more individuated vocal signals through volitional modulation of the voice. Identity cues encoded in volitional laughter may not be limited to individuals, but might also include group-related information.

Volitional displays are subject to a variety of cultural factors, including display rules (i.e. norms about the appropriateness of expressions) and language [1]. For instance, speech can communicate a wealth of information about speaker identity, like regional accent, especially when produced in a familiar language [13,14]. The movements of articulators that are produced while speaking differ systematically across languages and accents, allowing listeners to infer information about the cultural identity of the speaker [15]. It is, however, unclear to what extent cultural identity processing generalizes to other volitional vocalizations although it has been suggested that volitional laughter is likely to differ depending on the linguistic structure of the language spoken by the laughing person [16]. Languages differ in terms of articulatory settings such as the position of tongue, lips and jaw, which may shape articulation during the production of volitional nonverbal vocalizations including laughs. In the present study, we test whether listeners might make use of cross-linguistic differences when inferring a laughing person's cultural group membership, particularly from volitional laughter.

### (b) Identification of group membership from laughter

There are systematic differences across cultural groups in emotional expressive styles including nonverbal vocalizations [17]. These differences are sustained and potentially exacerbated over time as individuals learn expressive behaviours from their cultural environment. Culture-specific expressions thus exist around universally shared expression patterns [18]. The differences between the vocalizations of different groups are also notable to listeners, resulting in perceivers being more accurate in recognizing emotions from vocal expressions produced by individuals from their own cultural group as compared to others (recent meta-analysis: [19]). For instance, cross-cultural studies of emotion recognition found evidence for in-group advantage in recognition of emotions like happiness/joy from speech prosody.

The superior recognition of emotions from in-group vocalizations suggests that vocal expressions might even signal to listeners whether the vocalizing individual is an in-group member. To date, to our knowledge, only two studies have examined the identification of group membership from nonverbal vocalizations. Sauter [20] tested whether listeners could identify group membership from posed nonverbal vocalizations including laughs, produced by Dutch, British and Namibian speakers. Dutch listeners were asked to judge whether the individual who produced each vocalization was from The Netherlands, another European country or a country outside of Europe. The results showed that listeners were not able to accurately judge group membership, with especially high rate of confusions between Dutch and British vocalizations. In another study, Ritter & Sauter [21] tested whether group identity could be inferred from laughter specifically. The stimuli included both posed and spontaneous laughs, produced by Dutch, English, French, American, Japanese and Namibian speakers. Dutch participants were asked to judge the nationality of the laughing person in a six-way forced-choice task. Frequentist and Bayesian statistical analyses showed that listeners could not accurately identify group membership from laughter.

Several limitations of this work preclude drawing strong conclusions from these results. Firstly, the complexity of the group categorization tasks (e.g. in-group, a close out-group, a distant out-group; six different nationalities) may have hampered listeners' performance. Second, laughter type was not controlled in these studies. Given that spontaneous and volitional laughter are produced using different vocal production systems, accurate perception of group identity may depend on laughter type. Here, we test whether listeners can identify group membership from spontaneous and volitional laughter separately, and employ simple cultural in-group versus cultural out-group judgements.

### (c) Perception of positivity in laughter

In addition to differences in production, spontaneous and volitional laughter differ in terms of positivity. Spontaneous laughter is typically an uncontrolled reaction to outside events which includes hard-to-fake features (e.g. high oscillation rates of the intrinsic laryngeal muscles), while voluntary laughter is more easily inhibited or modified, reflecting a more deliberate communicative act like conveying polite agreement [3,10,16]. Spontaneous laughter might, therefore, be expected to sound more positive compared to volitional laughter. Indeed, previous research has found that spontaneous laughter is perceived as more positively valenced [22,23]. In the current investigation, we test whether this difference would generalize across cultures.

In addition to laughter type, group membership might also influence how positively laughter is perceived. Even for arbitrarily formed groups, people reliably evaluate in-groups more positively than out-groups [24,25]. The activation of in-group concepts exerts top-down positivity effects on judgements, including of emotional expressions [26]. Group perceptions might thus bias participants such that laughs *thought* to come from in-group members may be perceived as sounding more positive. Here, we, therefore, test whether laughs are perceived as more positive when the laughing person is an in-group member as compared to an out-group member.

### (d) The present study

In the present study, we sought to compare the accuracy of group membership identification for volitional laughter to that from spontaneous laughter, as well as to examine the positivity perception of laughter across cultures. We employed laughter clips that were spontaneously or volitionally produced by Dutch and Japanese individuals (spontaneous_prod_, volitional_prod_). Dutch and Japanese participants then listened to those clips and judged (i) whether the laughing person was from their own or another culture; and (ii) whether they perceived the laughter as spontaneous or volitional (spontaneous_perc_, volitional_perc_). They also rated the positivity of each laughter clip.

We first examined whether listeners could judge group membership of producers at better-than-chance levels. This allowed us to examine participants’ ability to differentiate in-group from out-group laughter, independently of laughter type. We then tested whether the accuracy of group membership identification would be higher for volitional compared to spontaneous laughter to test our prediction that performance would be better for volitional laughter. We further tested the prediction that spontaneous laughter would be rated as more positive than volitional laughter, and examined whether in-group laughs would be rated as more positive than out-group laughs. In all analyses, we separately examined judgements of laughter type based on production (spontaneous_prod_, volitional_prod_), as well as perception (spontaneous_perc,_ volitional_perc_). Perceived laughter types might be affected by different factors than produced laughter types, like familiarity with the out-group laughs. Including both measures allows us to understand differences in group identification and perceived positivity across spontaneously and volitionally produced laughs, as well as in relation to how spontaneous or volitional the sounds are.

## 2. Methods

### (a) Participants

The study used an opportunistic sample, collecting as many responses as possible. A total of 977 Dutch participants took part in the study at the science museum NEMO in Amsterdam, The Netherlands, during a two-week period in 2014. Participants were excluded from the present analysis if they were less than 18 years old (544 participants), were not Dutch (78 participants), did not complete the whole experiment (77 participants), reported visual/auditory impairments (four participants) or had lived abroad for more than six months (one participant). The remaining 273 participants (165 women, 108 men) had a mean age (*M*_age_) of 43.15 years (s.d._age_ = 9.84, range = 19–75 years old).

A total of 330 Japanese individuals participated in the study at Miraikan, National Museum of Emerging Science and Innovation in Tokyo, Japan, during a two-week period in 2016. Participants were excluded from the present analysis if they were below 18 years old (158 participants), did not complete the experiment (15 participants), had lived abroad for more than six months (14 participants), were not Japanese (four participants), reported visual/auditory impairments (two participants) or because of errors in the data log (six participants). The remaining 131 participants (71 women, 60 men) had an *M*_age_ of 36.92 years (s.d._age_ = 10.44, range = 18–68 years old).

### (b) Materials and procedure

#### (i) Stimuli

Spontaneous and volitional laughs were recorded in a sound-proof room. Individuals whose native language was Dutch/Japanese and who had never been diagnosed with any voice disorder were recruited for the recordings. Six Dutch (three women, three men; *M*_age_ = 34.83, s.d._age_ = 11.23, range = 23–55 years old) and six Japanese (three women, three men; *M*_age_ = 32.17, s.d._age_ = 11.46, range = 21–49 years old) speakers produced the laughter samples.

The spontaneous laughter clips were recorded while the participants laughed in response to self-selected funny videos. For the volitional laughter, participants were instructed to politely laugh at non-funny jokes told by a confederate. Participants could produce multiple laughter samples while watching a given video or in response to a bad joke. The production of spontaneous and volitional laughter production was blocked and the order randomized across participants. All stimuli were recorded using a Tascam DR-2d portable recorder sampled at a 44 kHz sampling rate (16-bit, mono). The laughter samples were cut into segments of bouts of laughter using Praat [27]. Laughs were selected based on having at least one breath group with introductory breaths excluded, maximum duration of 5 s, and no overlapping speech. In total, 795 laughter samples were collected (350 Dutch, 445 Japanese). Recordings were normalized to a peak amplitude of 0.95 Pa, and faded in and out with a co-sine squared ramp.

The main experiment was planned to run in science museums based on voluntary participation, requiring the experiment duration to be short. Consequently, a pilot study was conducted to select a small set of laughter clips to be included in the main experiment. Twenty Dutch (16 women, four men; *M*_age_ = 23.15, s.d._age_ = 2.87, range = 18–29 years old) and 18 Japanese participants (all women, *M*_age_ = 20.28, s.d._age_ = 1.04, range = 19–23 years old) were recruited for the pilot study. Dutch (Japanese) participants listened to all Dutch (Japanese) laughter clips. Participation in the pilot study was compensated with a monetary award or research credits. Participants were asked to answer two questions: ‘Do you think this was a genuine or a polite laugh?’ and ‘Did this laugh sound authentic or not?’ using yes/no response options. Sixteen clips (eight Dutch, eight Japanese; laughter type and gender balanced for each group) that were most accurately discriminated as spontaneous versus volitional and that were judged as most authentic were selected as stimuli for the main experiment (see the electronic supplementary material, table S1 for details). The acoustic characteristics of the laughter clips used in this study were extracted using Praat [27]. Summary statistics show that spontaneous laughs had higher rates of intervoicing interval, higher duration, increased F0, F1 and F2 means, lower amplitude variability, higher spectral centres of gravity and reduced harmonics-to-noise ratios, which is consistent with previous research [2,16,22]. The acoustic characteristics of the laughter clips are provided in the electronic supplementary material, table S2 and presented in the electronic supplementary material, figure S1. Examples of spontaneous and volitional laughter clips for each cultural group can be found at https://emotionwaves.github.io/laughter/.

#### (ii) Experimental procedure

The 16 laughter stimuli were presented to participants via headphones from a computer. On each trial, participants heard a laugh and were asked in a two-way forced-choice task (i) whether the laugh was spontaneous, which happens when someone finds something really funny (e.g. a hilarious joke) or volitional, which happens when someone is laughing to be nice even though they do not think something is funny (e.g. a joke that is not funny at all); and (ii) whether the laughing person was from their own or cultural group or foreign. Participants also rated the positivity of each clip on a 7-point Likert scale ranging from 1 (a little positive) to 7 (very positive). The scale was presented with accompanying smiley faces (see the electronic supplementary material, figure S2). The presentation order of the stimuli and questions was randomized separately for each participant. They could replay each stimulus as many times as they wanted.

## 3. Results

### (a) Data processing

We quantified participants' ability to recognize group membership using the sensitivity index d-prime. d-prime controls for individual biases in the use of a particular response, and is calculated as z-transformed hit rates minus false alarm rates [28]. Hit rates were calculated as the proportion of in-group laughter trials to which participants responded with their own culture, and false alarm rates as the proportion of out-group laughter trials responded to as own culture. This transformation was calculated for spontaneous and volitional laughter separately. Hit and false alarm rates with extreme values (i.e. 0 or 1) return an error when z-transformed. Those cases are commonly adjusted by replacing rates of zero with 0.5/*n* (0.5/*m*) and rates of 1 with (*n* − 0.5)/*n* ([*m* − 0.5]/*m*), where *n* (*m*) is the number of signal (noise) trials [29]. All values from the signal detection analysis are provided in the electronic supplementary material, table S3.

### (b) Identification of group membership from laughter

Kolmogorov–Smirnov tests indicated non-normality in the distribution of d-prime scores (*p*s < 0.05), preventing the use of *t*-tests. We, therefore, used one-sample Wilcoxon signed-rank tests to test whether Dutch and Japanese participants could accurately judge group membership from spontaneous and volitional laughter. The laughter type was assessed both based on how the laughter was produced (spontaneous_prod_, volitional_prod_) and how the listener categorized the laughter type (spontaneous_perc_, volitional_perc_). Spontaneously produced laughter was categorized correctly with high recognition rates (*n* = 2468, 76.36%) by Dutch (*n* = 1660, 76.01%) and Japanese (*n* = 808, 77.01%) participants. Similarly, correct percentages were high in categorization of volitionally produced laughter (*n* = 2369, 73.30%) for Dutch (*n* = 1558, 71.34%) and Japanese (*n* = 811, 77.39%) listeners. The inclusion of production- and perception-based laughter types created eight conditions: group of the listener (Dutch, Japanese) × laughter type (spontaneous, volitional) × laughter type categorization (production, perception). d-prime scores for each condition were tested against chance (random guessing denoted by a d-prime score of zero), using separate Wilcoxon signed-rank tests for each condition. We expected Dutch and Japanese listeners’ d-prime scores to be significantly higher than chance for each perceived and produced laughter type, Bonferroni corrected for multiple comparisons (*α* = 0.006 [0.05/8]). All analyses concerning group identity processing were conducted in JASP (JASP Team, 2020).

Table 1 presents d-prime values for each condition. The results show that both Dutch and Japanese participants could identify group membership at better-than-chance levels from both spontaneous and volitional laughter.

**Table 1.** D-prime scores indicating participants’ performance in judging group membership from laughter, tested against chance level (d-prime score of zero).

listener culture	laughter type^a^	*M* (s.d.)	median	*n*	*Z*	*r*	*p*
Dutch	spontaneous_prod_	0.59 (0.82)	0.67	273	19.197	1.16	<0.001
	volitional_prod_	0.53 (0.89)	0.67	273	17.194	1.04	<0.001
Japanese	spontaneous_prod_	0.40 (0.84)	0.48	131	3.466	0.30	<0.001
	volitional_prod_	0.38 (0.86)	0.48	131	3.362	0.29	<0.001
Dutch	spontaneous_perc_	0.52 (0.83)	0.48	270	1.138	0.07	<0.001
	volitional_perc_	0.52 (0.82)	0.59	267	1.103	0.07	<0.001
Japanese	spontaneous_perc_	0.37 (0.79)	0.25	129	4.680	0.41	<0.001
	volitional_perc_	0.41 (0.79)	0.43	128	5.040	0.45	<0.001

^a^Laughter type was categorized based on how speakers produced laughs (production, denoted _prod_), and how listeners categorized the laughter types (perception, denoted _perc_).

### (c) Comparison of group membership identification from spontaneous and volitional laughter

**Figure 1.**
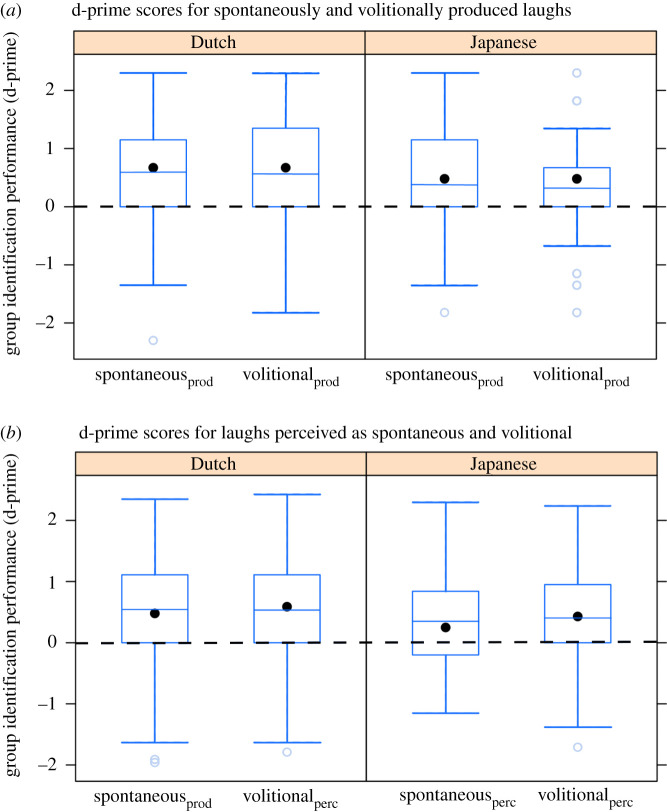
Boxplot of d-prime scores per laughter type showing Dutch and Japanese listeners' performance in identification of group membership from laughter. Black dashed line indicates the chance level. There was no significant difference in performance for Dutch or Japanese listeners across the laughter types. Black dots are medians, horizontal lines are means, box edges indicate the 95% confidence intervals for the medians, and the whiskers indicate minimum and maximum excluding outliers, which are marked with circles. (Online version in colour.)

In order to test whether listeners performed better at identifying group membership from volitional than spontaneous laughter, d-prime scores for the two laughter types were compared with paired samples Wilcoxon signed-rank tests. We expected d-prime scores, a sensitivity index of participants' ability to recognize group membership, to be significantly higher for volitionally produced as compared to spontaneously produced laugher, and for laughs perceived as volitional compared to laughs perceived as spontaneous. However, participants’ performance did not differ between spontaneous and volitional laughter: group membership identification performance of the Dutch (*Z* = −0.689, *p* = −0.491) and Japanese (*Z* = 0.056, *p* = 0.955) listeners did not differ between spontaneously and volitionally produced laughter. Similarly, listeners were not more accurate in identifying group membership from laughs perceived as volitional than from laughs perceived as spontaneous (Dutch: *Z* = −0.344, *p* = −0.731; Japanese: *Z* = −0.610, *p* = 0.542) (figure 1).

### (d) Testing the probability of the null hypothesis

Given the non-significant frequentist results, we additionally conducted Bayesian equivalent tests in order to probe the probability of the alternative and the null hypotheses. We compared the volitional d-prime scores to the spontaneous d-prime scores for Dutch and Japanese listeners in a Bayesian paired samples Wilcoxon signed-rank test with 500 iterations of data augmentation. The paired sample Bayesian Wilcoxon test estimates the effect size (*δ*) of how plausible the data is under the alternative versus the null hypothesis, resulting in a Bayes factor (BF). The Bayes factor BF_10_ quantifies the evidence that the data provide for H_1_ as compared to H_0_, while BF_01_ quantifies the evidence in favour of H_0_ as compared to H_1_. If BF_10_ is lower than 1, then the analysis provides evidence for the null hypothesis, while a BF_10_ that is larger than 1 means that there is evidence for the alternative hypothesis. A Bayes factor BF_10_ over 100 is considered ‘extreme evidence for the alternative hypothesis' [30,31]. We used the recommended default Cauchy distribution with a scale of 0 to 0.707 (*r* = √(0.5) = 0.707) as our prior distribution [32].

**Figure 2.**
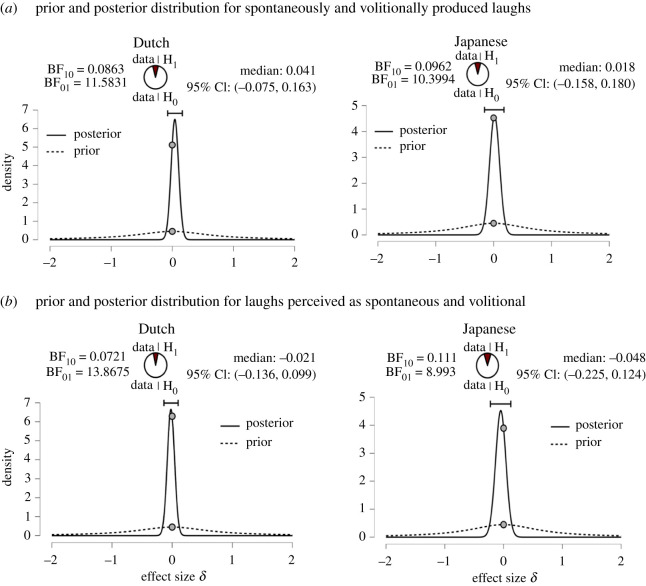
Inferential graphs of Bayesian statistics testing the probability of the alternative and null hypotheses. The prior distribution shows the distribution under the null hypothesis with performance at the chance level. The posterior distribution shows the distribution expected given the data. A score of zero on the *x*-axis indicates performance at the chance level. A BF_10_ lower than 1 provides evidence for the null hypothesis, and the higher the BF_01_ the higher the probability of the data occurring under the null hypothesis. For all conditions, data were more likely to have occurred under the null hypothesis than the alternative hypothesis. (Online version in colour.)

The null hypothesis—that listeners' performance in judging the group membership from laughter does not differ between the two laughter types—was supported over the alternative hypothesis. Specifically, for Dutch participants, the type of laughter did not make a difference to accuracy in group membership judgements when the laughter type was categorized based on production (spontaneous_prod_, volitional_prod_) median = 0.04, 95% confidence interval (CI) [−0.075, 0.163], BF_01_ = 11.583, *W* = 13.829 or perception (spontaneous_perc_, volitional_perc_) median = −0.02, 95% CI [−0.136, 0.099], BF_01_ = 13.868, *W* = 16.304.5, with a strong effect indicated in favour of the null hypothesis. The data from Dutch listeners was 11.583 and 13.868 times more likely to have occurred under the null hypothesis than the alternative hypothesis, respectively. Similarly, for Japanese participants, the accuracy of group membership judgements did not differ between the two laughter types, considered in terms of production median = 0.018, 95% CI [−0.158, 0.180], BF_01_ = 10.399, *W* = 2800 or perception median = −0.05, 95% CI [−0.225, 0.124], BF_01_ = 8.993, *W* = 3.402, with strong to moderate evidence in favour of the null hypothesis. This means that the data from Japanese listeners was 10.399 and 3.402 times more likely to have occurred under the null hypothesis than the alternative hypothesis, respectively. Inferential graphs can be found in figure 2.

### (e) Perceived positivity of laughter

Positivity ratings were normally distributed according to the Shapiro–Wilk test, allowing us to conduct pairwise *t*-tests comparing perceived positivity for spontaneous and volitional laughter. The results showed that both Dutch and Japanese participants rated spontaneous laughter as more positive than volitional laughter, both when the laughter type was categorized based on production and when it was categorized in terms of perception (table 2). Average positivity ratings for each clip are illustrated in the electronic supplementary material, figure S3.

**Table 2.** Paired sample *t*-test comparing perceived positivity across laughter types and cultures.

listener culture	laughter type categorization^a^	spontaneous		volitional		*t*-test per laughter type
		*M*	s.d.	*M*	s.d.	*t, p*
Dutch	production	4.91	0.73	3.29	0.81	32.199, <0.001
	perception	5.08	0.74	3.02	0.82	34.557, <0.001
Japanese	production	4.77	0.84	2.92	0.81	29.283, <0.001
	perception	5.00	0.84	2.66	0.64	33.540, <0.001

^a^Laughter type was categorized based on how speakers produced laughts (i.e. production), and how listeners categorized the laughter types (i.e. perception).

We also tested whether participants rated in-group laughs as more positive than out-group laughs. Pairwise *t*-tests showed that in-group laughs (*M* = 4.24, s.d. = 0.66) were evaluated as more positive than out-group laughs (*M* = 3.96, s.d. = 0.79) by Dutch listeners (*t*_272_ = 7.185, *p* < 0.001). However, Japanese listeners, on the contrary, rated laughs produced by in-group members (*M* = 3.69, s.d. = 0.79) as less positive than laughs produced by out-group members (*M* = 3.99, s.d. = 0.81; *t*_130_ = −6.161, *p* < 0.001). We then tested whether participants rated laughs as more positive when they judged the laughing person to be an in-group member, as compared with laughter from a perceived out-group member. Pairwise *t*-tests showed that laughs of perceived in-group members (*M* = 4.29, s.d. = 0.80) were rated as more positive than outgroup members (*M* = 3.81, s.d. = 0.91) by Dutch participants (*t*_266_ = 7.586, *p* < 0.001). There was no difference in positivity ratings between perceived in-group (*M* = 3.84, s.d. = 0.93) and out-group members (*M* = 3.84, s.d. = 0.84) for Japanese listeners (*t*_128_ = 0.127, *p* = 0.899).

## 4. General discussion

The present study demonstrates that listeners can infer whether a laughing person is from their own or another cultural group at better-than-chance accuracy levels based on only hearing a brief laughter segment. Contrary to prediction, we found no advantage for volitional laughter; neither frequentist nor Bayesian analysis yielded any support for the notion that participants would be better at identifying group membership from volitional as compared to spontaneous laughter. Finally, spontaneous laughter was rated as more positive than volitional laughter by both Dutch and Japanese listeners.

### (a) Group membership identification from laughter

Both Dutch and Japanese listeners were able to infer group membership from laughter. Our findings are in line with previous research showing that perceivers can identify group membership from speech segments [33] and language dialects [34]. Facial expressions also allow perceivers to infer the nationality of producers, even from visually similar groups like White Americans and Australians [35], and Japanese and Japanese-Americans [36]. Our study adds laughter to this suite of communicative signals by providing evidence that laughter can convey information about cultural group identity. Notably, accuracy levels are comparable across some of these domains. For instance, Walton & Orlikoff [33] found that listeners could correctly identify speakers' race from sustained vowel sounds with 60% accuracy in a two-way forced-choice task. In our study, the accuracy of group identification from laughter was 62% for Dutch and 55% for Japanese listeners. Identification accuracies are thus far from the ceiling, but clearly at better-than-chance levels. Our findings indicate that similarly to other types of communicative signals, cultural differences in how people laugh allow listeners to accurately infer whether a laughing person is from their own or another cultural group. The existence of such accents in nonverbal vocalizations also aligns with findings on superior emotion recognition from in-group compared to out-group nonverbal vocalizations [19].

Our results contrast with findings from previous studies, in which listeners have not been able to identify group membership from laughter at better-than-chance levels [20,21]. The current investigation employed a simpler task than those used in previous research. It is possible that the more challenging tasks used in previous studies was the reason why listeners were unable to discern group membership from laughter. The simple two-way in-group/out-group approach may be key to listeners being able to accurately tell whether a laughing person is a member of their own group. This would suggest that listeners are only able to infer rudimentary group information from laughter, but not information sufficient to making more complex judgements.

### (b) Judging group membership from spontaneous and volitional laughter

We found no difference in the accuracy of group membership identification from spontaneous versus volitional laughter. Differences in production mechanisms suggest that different types of information might be encoded in more or less reliable ways for the two types of laughter. Previous studies have shown that discrimination of speaker identity is superior from volitional laughter [7,8], suggesting that flexible modulation of the voice allows identity information to be encoded more reliably in volitional as compared to spontaneous laughter. The contrast between the results relating to individual and group identification might point to variability in laughter between individuals within a group potentially obscuring any group-related features relating to language. Our results, however, only address the perception side of differences between spontaneous and volitional laugher. We found that listeners do not perform better in group identification from volitional laughter, but we cannot speak as to whether the volitional laughs were more different between Dutch and Japanese individuals than were the spontaneous laughs. Given the small number of speakers and the necessary pre-selection of stimuli, only descriptive acoustic analysis was conducted in the current study. We selected the most distinctive spontaneous and volitional laughter clips based on a pilot study, resulting in 16 clips produced by eight speakers. Future research might use a larger number of laughs produced by more speakers, and directly test which acoustic properties of volitional and spontaneous laughter predict group membership identification.

### (c) Positivity, laughter type and group membership

We found that both Dutch and Japanese listeners judged spontaneous laughter to be more positive than volitional laughter. This aligns with previous research [22,23], providing evidence for the robustness of this finding across cultural contexts. We also found that in-group laughter was perceived as more positive than out-group laughter by Dutch listeners. However, such effect was not found for Japanese listeners. We speculate that these cultural differences in judgements of positivity of in- and out-group signals might be related to differences in self-enhancement across cultures. Motivations for positive self-views are more pervasive in individualistic cultures (such as the Dutch) than collectivistic cultures (such as the Japanese). This results in more positive evaluations toward groups to which an individual belongs in individualistic cultures [37]. For instance, North American students hold more positive attitudes towards students from their own university compared to students from other universities, while Japanese students do not show such a bias towards their in-group [38]. It is thus possible that the positivity bias towards in-group laughter in the present study may be owing to cultural differences in self-enhancement motivations.

### (d) Limitations and suggestions for further research

Our study has several limitations that merit consideration. Firstly, we recorded spontaneous laughter as a response to an amusing video and volitional laughter as a response to non-funny jokes. These clips might differ from laughter that occur in other situations that elicit spontaneous and volitional laughter, especially in more social contexts. Secondly, in real-life social interactions, laughs might be produced by a combination of spontaneous and volitional mechanisms. Future research should ideally include a more extensive set of stimuli that better capture the rich variability in spontaneous and volitional laughter. Relatedly, only the most distinguishable spontaneous and volitional laughter clips were included in the present study, based on pilot testing. This resulted in the exclusion of more ambiguous sounding laughs. Further work may use a larger set of laughter that varies in terms of differentiability.

## 5. Conclusion

The present study provides evidence for accurate group membership identification from decontextualized laughter. Moreover, our results suggest that laughter produced with volitional control might not confer an advantage for group membership inferences. Finally, we show that listeners across cultural contexts perceive spontaneous laughter as more positive than volitional laughter. Together, these findings add to the growing literature on laughter as a rich vocal signal that can be used by listeners to make a wide range of inferences about others, from their social relationships to their identity [8,39].

## Data accessibility

Data are available from the following repository: https://doi.org/10.21942/uva.14035865 [40].
